# The methods and techniques of identifying renal pedicle vessels during retroperitoneal laparoscopic radical and partial nephrectomy

**DOI:** 10.1186/s12957-019-1580-1

**Published:** 2019-02-22

**Authors:** Feiya Yang, Qiang Zhou, Xuesong Li, Nianzeng Xing

**Affiliations:** 1 Department of Urology, National Cancer Center/National Clinical Research Center for Cancer/Cancer Hospital, Chinese Academy of Medical Sciences and Peking Union Medical College, No. 17, Panjiayuan South Li, Chaoyang District, Beijing, 100021 People’s Republic of China; 2 Department of Urology, Zhongnan Hospital of Wuhan University, Wuhan, China; 3 Department of Urology, Beijing Chaoyang Hospital, Capital Medical University, Beijing, People’s Republic of China; 4 Department of Urology, National Urological Cancer Center, Peking University First Hospital, Institute of Urology, Peking University, Beijing, People’s Republic of China

**Keywords:** Laparoscopy, Renal pedicle, Nephrectomy, Partial nephrectomy, Retroperitoneal, Three-step method

## Abstract

**Background:**

Retroperitoneal laparoscopic radical and partial nephrectomy (RLRN and RLPN) have become the preferred modes of management for renal malignancy. One of the most critical steps in the RLRN and RLPN process is to seek and control the renal pedicle. The current study focuses on introducing methods and techniques that can help quickly and accurately identify the renal pedicle vessels during RLRN and RLPN.

**Methods:**

RLRNs and RLPNs were performed for 292 cases in our hospital from November 2014 to January 2017. Different measures were adopted to seek and manage bilateral renal pedicle vessels. All operation procedures were performed by the following three steps: dissection, opening, and clamping. For the left lateral, after the perirenal fat in the dorsal and lateral side was fully dissected, the kidney was pushed toward the ventral side. The renal artery was visible when opening the dense bulging connective tissue, which was located in the middle of the dorsal interior of the kidney. Then, the renal artery was clamped with a Hem-o-lok or the Bulldog clamp. For the right kidney pedicles, the inferior vena cava was first identified and then dissipated upward. When the inferior vena cava was not visible, it was often the location of the right renal artery. The treatment for the artery was the same as for the left renal artery. Relevant clinical characteristics of patients, such as operative time, intraoperative blood loss, and duration of postoperative drainage, were analyzed retrospectively. The three-step method of identifying renal pedicle vessels during retroperitoneal laparoscopic radical and partial nephrectomy was evaluated.

**Results:**

All operations were successfully accomplished with satisfying results, during which the artery could be controlled quickly, and no cases were converted to open surgery due to severe bleeding of renal pedicle vessels. There were no complications involving renal vessels during the entire study. The mean operative times were (81.9 ± 19.71) min and (88.2 ± 21.28) min for RLRN and RLPN, with an average intraoperative blood loss of (91.7 ± 47.10) ml and (62.4 ± 47.45) ml, respectively. The warm ischemia time for RLPN was (19.3 ± 5.6) min. The postoperative drainage-tube was removed within (4.5 ± 1.29) d (RLRN) and (4.6 ± 1.98) d (RLPN); the mean postoperative hospital stay times were (7.0 ± 2.4) d and (5.9 ± 1.98) d, respectively.

**Conclusion:**

The three-step method of identifying renal pedicle vessels during RLRN and RLPN is direct and feasible, and it may help simplify the operating procedure and improve the safety of the surgery. It may be of great practical application value in the clinical field.

**Electronic supplementary material:**

The online version of this article (10.1186/s12957-019-1580-1) contains supplementary material, which is available to authorized users.

## Background

Laparoscopic radical nephrectomy (LRN) or laparoscopic partial nephrectomy (LPN) has gradually become a standard surgical procedure for the treatment of renal malignancy [1–5]. One of the most critical steps in the LRN and LPN process is to seek and control the renal pedicle. If the renal vessels cannot be quickly and accurately found and correctly processed during the operation, the risk of pedicle injury will be increased, directly leading to massive hemorrhage and open conversion [6]. In recent years, retroperitoneal laparoscopic techniques have been increasingly used in urological surgery, and how to successfully deal with these important procedures has become crucial. This study focuses on introducing our experiences of a three-step method of identifying and handling the renal pedicle vessels in retroperitoneal laparoscopic nephrectomy and partial nephrectomy and explores its safety and effectiveness.

## Methods

### Patients

From November 2014 to January 2017, 93 cases of retroperitoneal laparoscopic radical nephrectomy (RLRN) and 199 cases of retroperitoneal laparoscopic partial nephrectomy (RLPN) were performed with satisfactory results in our hospital, which included 66 male and 27 female patients in the RLRN group as well as 119 male and 80 female cases in the RLPN group. The operation procedures were performed by a single experienced surgeon (Nianzeng Xing), and the procedures were all the same. The mean age of the patients was (56.1 ± 11.18) years and (53.4 ± 12.34) years for the RLRN and RLPN group, respectively. In total, 46 patients underwent surgery on the right side and 47 on the left side in the RLPN cohort, and 95 cases underwent operation on the right side and 104 patients on the left side in the RLPN group. All patients were evaluated with B-ultrasound, renal spiral computed tomography (CT) scanning, and routine laboratory tests before surgery. Patients with the diagnosis of renal vein thrombus or lymph node metastasis were not included in our study. This study was approved by the Research Ethics Committee in our hospital (China) and every patient signed the informed consent before operation.

### Patient positioning and trocar location

After inducing general anesthesia with tracheal intubation, all patients were placed in a full lateral decubitus position with a raised waist bridge and received the retroperitoneal operation procedures. First, a 1.5–2 cm skin incision (point A) was made below the twelfth rib arch at the posterior axillary line, after which a large vascular clamp was used to bluntly separate the muscular layer and lumbar fascia. Then, the index finger was inserted into the gap, and the peritoneum was pushed forward. A balloon dilator was imbedded, and 500–1000 ml of air was injected to expand the retroperitoneal space for 3 min. Subsequently, under the guidance of the finger, a 5-mm trocar was placed through point A under the costal margin of the anterior axillary line (point B), and a 10-mm trocar was placed 2 cm above the iliac crest of the middle axillary line (point C). A supplementary 5-mm port could be positioned under point B during the operation if necessary. The incision was temporarily sutured in a whole layer with No. 10 silk thread to avoid gas leakage after placement of the 10-mm port. The 30° laparoscope was placed at point C, and point A and point B were used for relevant laparoscopic instruments. The retroperitoneal cavity was inflated with CO_2_, and the pressure was maintained at 12–15 mmHg during the operation.

### Three-step method to renal vessels

The methods for left and right kidney pedicles were different; however, all processes were implemented by the following main three steps (Additional files 1 and 2).

For the left renal pedicle:

Step 1: Dissection. After entering the retroperitoneum, the extraperitoneal fat was cleared adequately, and Gerota’s fascia was opened. Then, the perirenal fat was fully cleared, and blunt separation between the posterior renal fascia and lumbar fascia was applied to fully reveal the anterior space of the psoas muscle. Then, the lateral and dorsal side of the kidney was dissected, ranging up to the diaphragm and down to the lower pole of the kidney. The renal pedicle was located in the middle of the dorsal interior of the kidney, 2–4 cm below the medial arcuate ligament of the diaphragm. Seeking the kidney pedicle should be performed in this area.

**Fig. 1:**
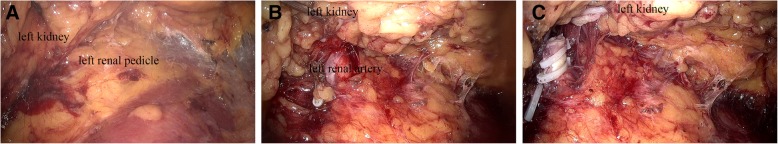
**a**, **b** The kidney is pushed to the ventral side, and dense bulging connective tissue can be seen in the middle of the dorsal interior side of the kidney as well as the arterial pulsation. The left renal artery can be exposed by opening the connective tissue. **c** Three Hem-o-lok clips were used to ligate the artery with two clips on the proximal side and one on the distal side

Step 2: Opening. Our experience involves using the vascular clamp to move the kidney toward the ventral side. In the middle of the dorsal interior of the kidney, dense bulging connective tissue can be seen, and the pulsation of the renal artery can also usually be clearly identified (Fig. 1a). When the connective tissue was opened by the blunt and sharp dissection, the renal artery was visible immediately (Fig. 1b). Then, a right angle clamp was used to bluntly dissect the renal artery toward the aorta until 2–3 cm of the renal artery was revealed.

Step 3: Clamping. A small amount of tissue should be reserved on the surface of the renal artery to reduce the chance of clip loss or injury of the renal artery. If the patient received RLRN, three Hem-o-lok clips were used to ligate the artery by cutting it with two clips on the proximal side and one on the distal side (Fig. 1c). Then, the abdominal aorta was slightly dissected in front of the upper and lower position of the renal artery, after which the renal vein could be exposed and handled consistent with the renal artery. If the left renal vein was difficult to find, the reproductive vein could be found first and act as a mark. Along this mark, the separation was performed, and the renal vein could be seen.

**Fig. 2:**
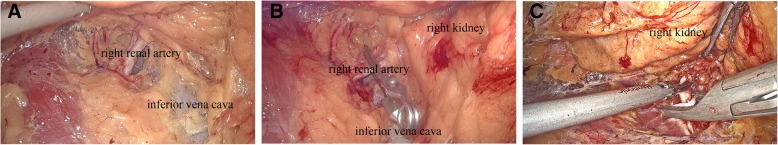
**a** The right renal artery is always located where the inferior vena cava is not visible. **b** A Bulldog clamp was applied to clamp the artery in retroperitoneal laparoscopic partial nephrectomy. **c** The curved tip of the Hem-o-lok should be placed toward the lens as much as possible in case of clamping other tissues

For the right renal pedicle:

Step 1: Dissection. The extraperitoneal fat, Gerota’s fascia, and perirenal fat were managed the same way as on the left side. As the inferior vena cava is easier to orient, it should first be dissected upward until arriving at the place where the inferior vena cava is not visible, which is often the location of the right renal artery (which we define as Xing’s rule; it is one of our important views and has been put into clinical practice) (Fig. 2a).

Step 2: Opening. The right kidney was moved fully toward the ventral side. In the ventral side of the right renal artery, the connective tissue between the kidney and the inferior vena cava was dissected and then opened (where the inferior vena cava was not visible), after which the right renal artery could be seen.

Step 3: Clamping. Before dealing with the right renal vein, the angle formed by the right renal vein and the inferior vena cava should be revealed completely in order to prevent the inferior vena cava from being injured. If the patient received RLRN, the method to manage the right pedicles was the same way as for the left.

After the renal pedicle was successfully processed, the ureter dissection was routinely performed and transected with a Hem-o-lok and harmonic scalpel. Then, the dissection was carried out at the ventral and medial side of the kidney, from the lower pole to the upper pole to complete the nephrectomy. The specimen was bagged and extracted via an oblique incision at the lower abdomen. When the drainage tube was placed into the retroperitoneal cavity, the incisions were closed, and the operation was finished.

**Fig. 3:**
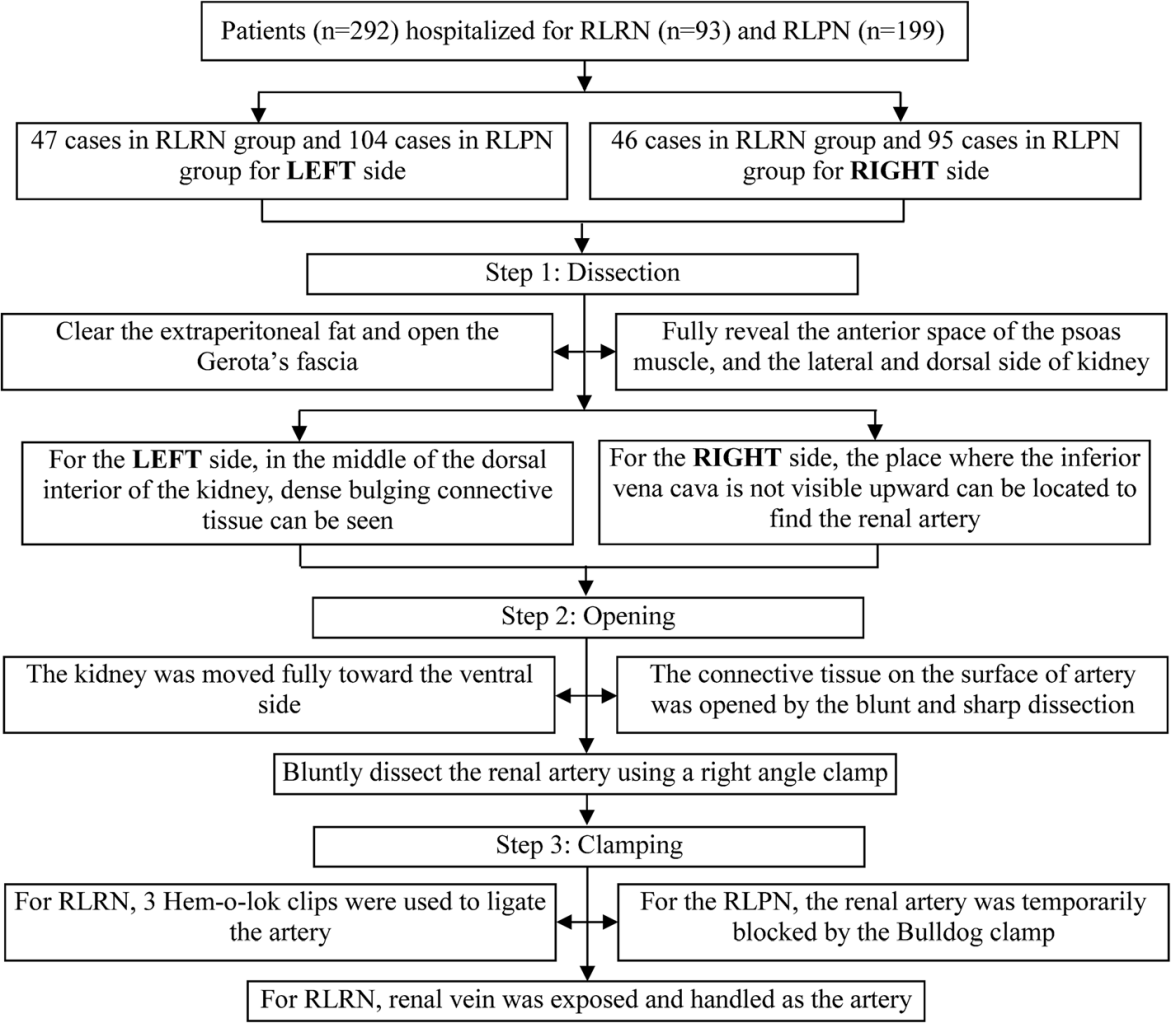
The line diagram of the steps of the procedure. RLRN, retroperitoneal laparoscopic radical nephrectomy; RLPN, retroperitoneal laparoscopic partial nephrectomy

For the RLPN, the procedure was the same as the RLRN before handing the vessels. After adequately separating the renal artery, the renal artery was temporarily blocked by the Bulldog clamp and should be controlled within 30 min (Fig. 2b). In addition, renal veins should also be clamped to prevent blood from counterflow when the tumor mass is located adjacent to the renal hilum. Then, the tumor was removed and the renal incision was closed within 30 min. (Fig. 3).

## Results

Appropriate handing of the renal pedicle is a crucial step in the RLRN and RLPN process because the majority of conversions to open surgery are caused by renal pedicle hemorrhage. By following the three steps, all surgical operations were successfully accomplished with satisfying results. As shown in Table 1, none of the 292 cases received conversion to open surgery due to the injury of the renal pedicle, and no case experienced complications of renal vessels. The mean operative times were (81.9 ± 19.71) min and (88.2 ± 21.28) min for RLRN and RLPN, with average intraoperative blood loss of (91.7 ± 47.10) ml and (62.4 ± 47.45) ml, respectively, of which the shortest case was available in only 14 min. The warm ischemia time of RLPN was (19.3 ± 5.6) min. The postoperative drainage tube was removed within (4.5 ± 1.29) d (RLRN) and (4.6 ± 1.98) d (RLPN). The mean postoperative hospital stay times were (7.0 ± 2.4) d (RLRN) and (5.9 ± 1.98) d (RLPN). No complications occurred due to the failure of the three-step method during the surgeries. The rate of minor postoperative complications (such as hypertension, fever, and pain of incision) in this series was 12.9% (RLRN) and 10.0% (RLPN), and the most important result was that no cases experienced severe complications.

**Table 1:** Clinical characteristics of the patients

	Surgical procedure	
	RLRN (*N* = 93)	RLPN (*N* = 199)
Age, years	56.1 ± 11.18*	53.4 ± 12.34
Tumor size, cm	5.5 ± 1.72	3.6 ± 1.52
Gender		
Male, *n* (%)	66 (71.0)	119 (59.8)
Female, *n* (%)	27 (29.0)	80 (40.2)
BMI, kg/m^2^	25.3 ± 3.82	25.6 ± 3.91
Laterality		
Right, *n* (%)	46 (49.5)	95 (47.7)
Left, *n* (%)	47 (50.5)	104 (52.3)
Converted to an open surgery, *n*	0	0
Operative time, min	81.9 ± 19.71	88.2 ± 21.28
Warm ischemia time, min	–	19.3 ± 5.6
Estimated blood loss, ml	91.7 ± 47.10	62.4 ± 47.45
Drainage-tube time, days	4.5 ± 1.29	4.6 ± 1.98
Postoperative hospital stay, days	7.0 ± 2.40	5.9 ± 1.98
Overall complications, *n* (%)	12 (12.9)	20 (10.0)
Vascular injury	0	0
Hypertension	2	3
Fever	3	5
Pain of incision	5	8
Urinary tract infections	2	4

*Abbreviations*: *RLRN* retroperitoneal laparoscopic radical nephrectomy, *RLPN* retroperitoneal laparoscopic partial nephrectomy, *BMI* body mass index

*The value = mean ± standard deviation

## Discussion

In recent decades, laparoscopic techniques have made great progress in urological surgery. With improvements of optics, electronics, endoscopic TV monitoring systems, and surgical instruments, laparoscopic surgery has been widely applied and popularized in urology. Since Clayman et al. first reported laparoscopic nephrectomy in 1991 [7], the procedure has gradually become a standard surgical model for the treatment of renal failure, renal malignancy, and other kidney diseases [1–3]. It is generally believed that laparoscopic radical and partial nephrectomy can be performed through both transperitoneal and retroperitoneal approaches, and each approach has its own advantages and limitations. Fan et al. reported that it took a shorter time for RLRN than for TLRN (transperitoneal laparoscopic radical nephrectomy) to control renal vascular and that the operating time for RLPN was shorter than that for TLPN (transperitoneal laparoscopic partial nephrectomy). When compared with the transperitoneal approach, the retroperitoneal approach was faster and equally safe for appropriately selected patients, especially those with posteriorly located renal tumors [8]. Wright and Porter and Ng et al. stated that it was more convenient to reach the kidney and its pedicle via the posterior abdominal cavity since there was little interference from the abdominal organs. Furthermore, most of the renal artery was located behind the renal vein, which made the renal artery more directly exposed and identifiable compared with the transperitoneal procedure [9, 10]. Of course, the transperitoneal approach can provide more space than the simple retroperitoneal approach and make the procedures easier especially when a complicated situation is confronted, such as an anteriorly located tumor [11].

As for additional strategies for nephrectomy, there are reports regarding laparoendoscopic single-site surgery (trans-umbilical and lumber) [12, 13], transumbilical multiport laparoscopic nephrectomy with specimen extraction through the vagina [14], laparoendoscopic single-site transvaginal nephrectomy [15, 16], and hybrid transvaginal NOTES nephrectomy [17]. The authors declared that these relatively novel operation modes were technically challenging but feasible and may be performed safely. This progress could further improve the cosmetic effects and decrease the risk of incisional hernia formation. While the existence of these methods is valuable, they may depend more on advances in equipment. In our opinion, the kind of operation that is adopted depends on the complexity of the renal tumor, personal skills and habits, and past surgical history of patients. The current study focused on our three-step method for controlling renal pedicle vessels during RLRN and RLPN.

Whether it is a left or right renal operation, the key and first step are to search for and deal with the renal artery. After finding the renal artery and clamping it with Hem-o-lok, the safety of the operation is largely guaranteed, and the surgeon will become much calmer when he or she handles the renal vein. Previous retroperitoneal techniques usually dissected, ligated, and divided renal vessels after the entire mobilization of the kidney (dorsal and abdominal side, lateral and medial side, upper and lower pole) [18, 19]. The renal pedicle was usually located mainly through landmarks, including the psoas muscle, the median arcuate ligament, and the diaphragm [20]. The control and division of the renal vessels using the linear cutting stapler was considered standard practice by most surgeons [21]. In the current study, we used a three-step approach during which only the dorsal and lateral side of the kidney needed to be dissected at the beginning, and this could facilitate early control of the renal pedicle. The rule of finding the left renal artery was that, after fully dissociating, the kidney was pushed to the ventral side, and dense bulging connective tissue could be seen in the middle of the dorsal interior side of the kidney as well as the arterial pulsation. After opening the connective tissue, the left renal artery could be exposed and corresponding treatment was implemented. As for the right renal artery, we first found the inferior vena cava and then dissipated upward along the inferior vena cava. When the inferior vena cava was not seen, it was often the location of the right renal artery. In the process of seeking the renal artery, the nameless venule was visible both in the left and right side, which was also a sign. Sometimes, the vascular condition was complicated; when it was difficult to find the renal artery, intraoperative ultrasound could be used [22]. When separating the renal artery, it was necessary to operate along the longitudinal axis of the artery. Lateral separation may cause damage to the surrounding tissue or vessel tearing. In the process of separation, the outer vascular sheath of the renal artery should be opened, and there was a distinct anatomical level between the vascular sheath and the arterial wall, which was easy to separate when the right plane was reached.

As for the laparoscopic nephrectomy realized through the complete vaginal approach or hybrid transvaginal NOTES [15–17], we think that the three-step method could be applicable in these cases. However, it will be more difficult for these cases than our reported retroperitoneal approach due to the problems caused by the surgical instruments and visual angles. However, for the left side, it will be easy to find the reproductive vein that acts as a mark, along which the separation is performed and the renal pedicle will be seen soon. Similarly, for the right side, it may be relatively easy to dissect the inferior vena cava first and then locate the place where the inferior vena cava is not visible to find the right renal artery. It may be easy to find and dissect the left reproductive vein and inferior vena cava through the complete vaginal approach.

Other methods to handle the renal pedicle have also been reported. Yuan et al. reported a lower pole approach for the management of renal vascular pedicle in retroperitoneal laparoscopic radical nephrectomy. Following working place establishment, the anterior and posterior renal fasciae 3.0 cm below the lower pole were transected and the ureter was mobilized. Then, the renal vein was dissected preliminarily to expose the renal artery. This method may be more suitable for obese patients, renal pedicular adhesion, and large tumors [23]. However, more attention should be paid to ureter dissection because the ureter is very sensitive to ischemia that may easily lead to stenosis and necrosis. Additionally, the renal vein may not easily be found preliminarily to expose the renal artery, especially on the right side. Zhang et al. and Tunc et al., respectively, introduced the transperitoneal approach of direct lateral access to the renal artery and direct upper kidney pole access of the renal pedicle for laparoscopic partial or radical nephrectomy [24, 25], and this can help deal with some complicated situations manifested as anteriorly located large tumors that are not easily performed through a retroperitoneal approach. On the other hand, the risk of superior mesenteric artery injury would be a fatal mistake for the patient [19], and it is not easy to control the renal artery. Thus, we should fully consider the patient’s condition and the advantages and disadvantages of various surgical methods and elect the most appropriate method to maximize the benefits of patients.

It has been reported that the incidence of renal vascular complications in laparoscopic nephrectomy is 0.4 to 3.5% [26, 27]. Right renal vessels are adjacent to the inferior vena cava, and the left renal vein always has many small branches. No matter whether the large vessels or the small branches are injured during the process of treating the renal vessels, it may lead to massive bleeding, unclear surgical field, and prolonged operation time. In addition, renal vascular variability should be taken seriously during surgery because abnormal renal vessels can be present unilaterally or bilaterally. Variations of the renal artery are more common than those of the kidney vein, but the renal vein and artery are rarely aberrant together [28, 29]. Therefore, the amplification function of laparoscopy should be should be placed on careful separation when handing the renal pedicle. We should seriously look at the preoperative CT or CT angiography (CTA) to identify aberrant vessels, even if there is no clear manifestation of mutative renal vasculature by the normal imaging examination before surgery. If the renal artery diameter is found to be thinner than normal in the surgery, it is necessary to pay more attention to identify if there are other arterial branches. During the procedures, there were no complications of vessels using the three-step method, which may be because all the operations were performed by a single experienced surgeon (Nianzeng Xing).

Many instruments are used to control renal pedicles, of which endoscopic gastrointestinal anastomosis (Endo-GIA) vascular staples and Hem-o-lok clips are most frequently applied [30, 31]. The characteristics of the former are that the length of arteries and veins only need to be dissociated approximately 1 cm for manipulation, and the side far from the visual field of vessels need not be completely freed. However, its deficiencies are high price, large volume, occupation of certain operating space, and the possibility of accidentally injuring the vena cava and causing an arteriovenous fistula [32–34]. In recent years, the Hem-o-lok has been increasingly applied for its effective clamping of renal vessels, increased safety, clear surgical field, and relatively low price [32, 33, 35]. Casale et al. retrospectively reviewed 31 laparoscopic nephrectomies in their department, with exclusive use of Hem-o-lok clips to control the renal pedicle. As a result, there were no transfusions, open conversions, or complications related to the use of Hem-o-lok clips. No renal vessel injuries and no cases of clip dislodgement, bleeding, or slippage were recorded. Moreover, a meaningful reduction in the cost per procedure was achieved by using clips [21]. Ping et al. also reported roughly the same results [35]. In the current study, all cases were treated with Hem-o-lok, achieving adequate vascular control during the procedure of RLRN, and no relevant vascular complications occurred. However, several points should be taken into consideration during the using of Hem-o-lok, of which we must confirm that the curved tip of the clip is toward the lens, and vessels should be dissected completely in case of clamping other tissues (Fig. 2c). If the renal vein is too wide to be completely encompassed by the Hem-o-lok, the blood vessels can be initially ligated with silk thread and then treated with the Hem-o-lok.

There were several limitations of our study. First, it was not a control study, and this lessened the strength of our study. In this respect, a retrospective or prospective controlled study should be performed to further verify the advantage of our three-step method. Second, it was a single-center study, and all the operations reported here were performed by a single experienced surgeon (Professor Nianzeng Xing). The results, i.e., that no complications related to renal vessels occurred, may not be broadly representative. Third, due to the different anatomical position and change, the three-step method could not be used in some transperitoneal approaches. For large tumors located in the dorsal hilum, the three-step method should be applied combined with other methods.

## Conclusions

In summary, RLRN and RLPN are safe and reliable procedures for the treatment of renal failure, renal malignancy, and other kidney diseases, and the key procedure of retroperitoneal laparoscopic radical nephrectomy or partial nephrectomy is the management of the renal pedicle. The three-step method used to identify renal pedicle vessels during RLRN and RLPN is direct and feasible, and it may help simplify the operating procedure and improve the safety of the surgery. It can be of great practical application value in the clinical field.


Additional file 1:Video for surgical steps of left side. (WMV 254275 kb)



Additional file 2:Video for surgical steps of right side. (WMV 221719 kb)


## Abbreviations

BMI: Body mass index
RLPN: Retroperitoneal laparoscopic partial nephrectomy
RLRN: Retroperitoneal laparoscopic radical nephrectomy
